# Intimate Relations: Molecular and Immunologic Interactions Between *Neisseria gonorrhoeae* and HIV-1

**DOI:** 10.3389/fmicb.2020.01299

**Published:** 2020-06-03

**Authors:** Furkan Guvenc, Rupert Kaul, Scott D. Gray-Owen

**Affiliations:** ^1^Department of Molecular Genetics, University of Toronto, Toronto, ON, Canada; ^2^Department of Medicine, University of Toronto, Toronto, ON, Canada; ^3^Department of Immunology, University of Toronto, Toronto, ON, Canada; ^4^Division of Infectious Diseases, University Health Network, Toronto, ON, Canada

**Keywords:** *Neisseria gonorrhoeae*, HIV-1, infectious synergy, co-infection, sexually transmitted infection, sexually transmitted disease

## Abstract

While the global incidence of human immunodeficiency virus (HIV-1) remains well above UNAIDS targets, sexual transmission HIV is surprisingly inefficient. A variety of host, viral and environmental factors can either increase HIV-1 shedding in the infected partner and/or increase mucosal susceptibility of the HIV-1 uninfected partner. Clinical and epidemiological studies have clearly established that *Neisseria gonorrhoeae* substantially enhances HIV-1 transmission, despite it not being an ulcerative infection. This review will consider findings from molecular, immunologic and clinical studies that have focused on each of these two human-restricted pathogens, in order to develop an integrative model that describes how gonococci can both increase mucosal shedding of HIV-1 from a co-infected person and facilitate virus establishment in a susceptible host.

## Introduction

It is remarkable to consider that human immunodeficiency virus type 1 (HIV-1) has caused a global pandemic despite being quite poorly transmissible, with the chance of an HIV-infected, treatment-naive individual passing the virus to their partner through penile-vaginal sex being a fraction of one percent (Royce et al., 1997; Galvin and Cohen, 2004). The explanation for this apparent contradiction is that multiple factors can enhance the likelihood of transmission, including the type of mucosa exposed to virus during sex, the viral titer in the anogenital secretions of the infected partner, and the presence/number of CD4 receptor-expressing target cells in the mucosa of the uninfected partner (Royce et al., 1997; Ferreira et al., 2014). When viewed through this lens, it becomes clear why sexually transmitted infections (STIs) might facilitate HIV-1 transmission, either directly through their effect on viral replication or indirectly through the inflammation-mediated recruitment of lymphocytes and/or disruption of the mucosal barrier. While a variety of co-infections can influence HIV-1 transmission (Fleming and Wasserheit, 1999; Kaul et al., 2008), this review will focus on the effects of *Neisseria gonorrhoeae*, both because gonococcal infection markedly enhances HIV-1 transmission at an epidemiological level (Fleming and Wasserheit, 1999; Cohen, 2012) and because a number of studies have explored the molecular and immunologic aspects of this synergy. When these studies are considered together, the complexity of the association between these pathogens starts to become clear, even though there are apparently contradictory findings depending upon the models used to explore this pathogen interaction. Herein, we will review the replication cycle of HIV-1 and consider the possible impact of *N. gonorrhoeae* infection. Then, we will attempt to integrate these research findings to generate a model that can be used to understand the outcome of this common co-infection, providing a framework to direct future research and a guide for ongoing attempts to intervene.

## HIV-1 Infection

Despite significant global efforts to control HIV-1 transmission, the virus continues to be a scourge, particularly in high risk populations that include men who have sex with other men (MSM) and female sex workers. Acute HIV-1 infection is characterized by a transient period of very high titer viremia that lasts for just a few weeks, which is followed by a chronic infection phase characterized by a much lower viral load in both blood and anorectal secretions once a host adaptive immune response has been generated. However, HIV-1 infection is lifelong, due in equal parts to the virus’s ability to integrate its genome into the chromosomal DNA of long-lived CD4+ memory T cells where it can reside undetected, while simultaneously evading the adaptive response during active replication by mutating its genome at a phenomenal rate [4 × 10^–3^ per base per cell, the highest for any described biological entity (Cuevas et al., 2015)]. For the latently infected cell, subsequent immune activation driven by infectious or other inflammatory stimuli will cause the virus to exit latency and initiate active replication resulting in viral propagation and eventual death of the infected cell. Over an average of 8–10 years, a combination of active cellular viral replication and bystander CD4+ T cell death leads to gradual depletion of blood CD4+ T-cell numbers, along with persistent immune activation and CD8+ T cell exhaustion and dysfunction, with irreparable damage to the structure of secondary lymphoid organs preventing recovery of depleted CD4+ T-cell counts. Once the blood CD4+ T cell count falls below ∼200 cells/ml, this is now classified as acquired immunodeficiency syndrome (AIDS), and the immune system is now unable to protect the infected individual against a variety of opportunistic infections and neoplasms [for review see Langford et al. (2007)].

Fortunately, HIV-1 is now a manageable chronic infection thanks to the development of antiretroviral compounds that target multiple different points of the viral life cycle. Combination antiretroviral therapy (cART) utilizes a combination of several of these compounds (generally 2–3 separate medications) to simultaneously block multiple stages of the viral life cycle and permit recovery of the immune system. Successful treatment suppresses virus replication to virtually undetectable level, restoring an individual’s life expectancy to near-normal (Arts and Hazuda, 2012). However, while effective at preventing HIV-1 replication, these medications are not a cure since the latent HIV reservoir remains unaffected by the treatment. Because of this, interruption of therapy consistently and predictably leads to the rebound of viral replication in blood, and the patient will progress to AIDS if medication is not continued. However, while the infected individual cannot be cured, the remarkable efficacy of cART is such that HIV-infected individuals who are compliant with their medication and maintain a suppressed viral load are considered to be non-infectious to their sexual partners (Cohen et al., 2016; Rodger et al., 2019).

By halting transmission, cART has contributed to a slow reduction in the global incidence of new HIV-1 infections (UNAIDS, 2019). However, the administration of therapy is costly, and some HIV-1 infected individuals do not have access to these drugs – this is particularly true within marginalized key populations such as MSM and female sex workers (Levi et al., 2016; Bain et al., 2017). According to UNAIDS (UNAIDS, 2019), there were 37.9 million people living with HIV-1 globally in 2018, with 23.3 million (61%) of these individuals on antiretroviral therapy. Indeed, the combination of ongoing HIV transmission by untreated (or inappropriately treated) individuals, together with the greatly enhanced survival of infected individuals taking antiretroviral treatment (ART), means that there is a steady increase in the global prevalence of HIV-1 each year; globally, with a 23% increase between 2010 and 2018 (UNAIDS, 2019). The clear message is that the only way to reduce the global burden of HIV-1 is to develop new and better interventions to reduce HIV-1 transmission and/or to develop feasible strategies for HIV cure.

## HIV-1 Transmission

Human immunodeficiency virus type 1 transmission risk varies greatly between populations, with heterogeneity linked to multiple behavioral and biological factors that include socioeconomic status, race, sexual partner numbers and patterning, mucosal site of exposure, circumcision status and contraceptive use, the presence of anogenital infections and the mucosal microbiome. Exposure of the anorectal mucosa to HIV through receptive anal intercourse remains the highest risk sexual behavior for HIV-1 transmission (Patel et al., 2014), with an incidence that averages 138 per 10,000 exposures. In comparison, the rates of transmission during penile-vaginal sex are approximately ten-fold lower, averaging 8 and 4 cases per 10,000 coital acts for male-to-female and female-to-male transmission, respectively (Patel et al., 2014). However, clinical and epidemiological studies have clearly established that HIV-1 transmission risk is substantially and consistently increased in the presence of certain co-infecting bacterial, viral and fungal pathogens (Galvin and Cohen, 2004) or inflammatory mucosal microbiota (Atashili et al., 2008; Mirmonsef et al., 2012; Schellenberg et al., 2012). In each case, the mucosal immune milieu is altered in response to these microbial challenges in a way that damages epithelial integrity and enhances HIV-1 shedding (in the infected partner) and access to preferred viral target cells in the uninfected partner, namely activated mucosal CD4^+^ helper T-cells.

**FIGURE 1:**
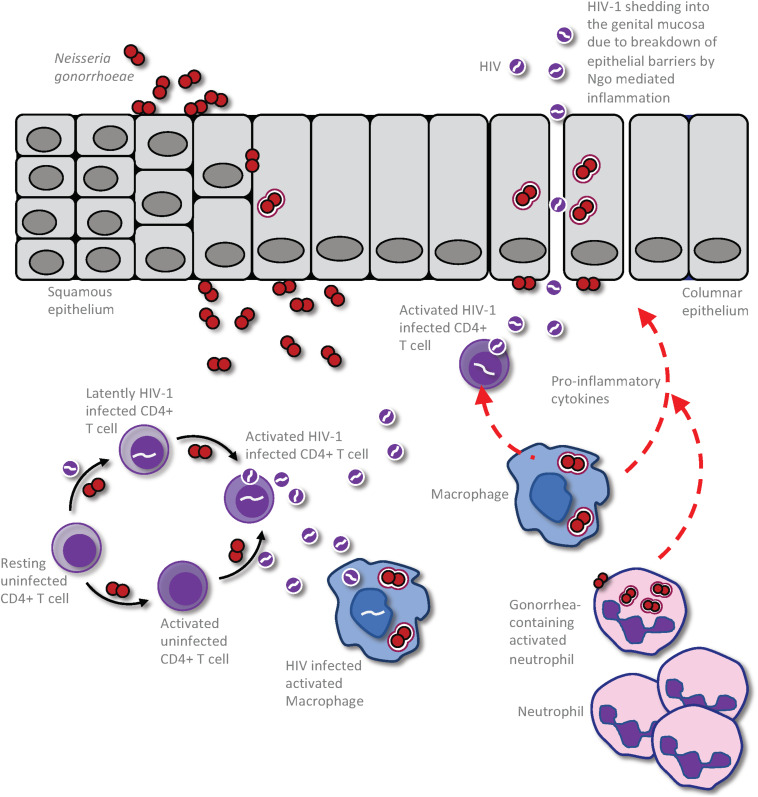
Impact of *Neisseria gonorrhoeae* infection on HIV production and shedding in an already HIV infected person. The presence of the gonococci within the urogenital tract results in an exuberant neutrophil response that is characterized by increased presence of pro-inflammatory cytokines and chemokines. This results in the recruitment of infected CD4 T-cells to the area of gonococcal infection, leading to activation of the latent HIV and release of virions to the genital microenvironment. This event could also result in infection of HIV-uninfected but gonococcal-activated CD4 T-cells and further propagation of the virus within the patient. Macrophage and dendritic cells (DCs) may also take up the virus to be presented to CD4 T-cells in distal lymphoid organs, increasing the efficiency of CD4 T cell infection and causing further propagation of infection, ultimately leading to destruction of secondary lymphoid organs. Following gonococcus-induced disruption of the epithelial barrier, either directly by the bacterium or through the inflammatory milieu, the cell free HIV particles may be released freely into the genital lumen to increase infectious potential of this HIV infected individual.

**FIGURE 2:**
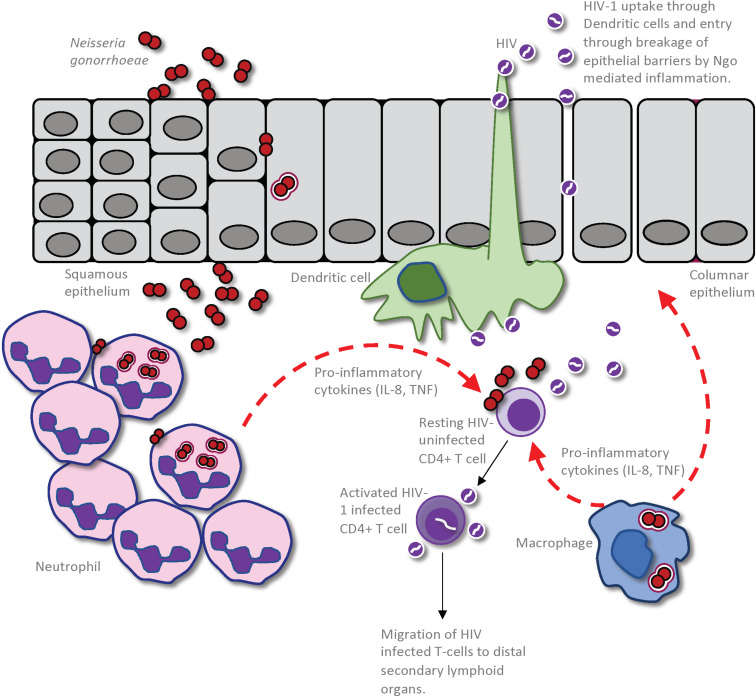
Impact of *Neisseria gonorrhoeae* infection on establishment of HIV infection in a seronegative individual. The gonococcus may cause an increased susceptibility to HIV infection through a combination of the inflammation-induced damage of the epithelial barrier and increased inflammation within the genital microenvironment. This allows enhanced entry of HIV into the submucosa, either through dendritic cells (DCs) that are sampling the mucosal environment or through disrupted epithelium. Given that the gonococci cause an intense localized inflammation, as evidenced by an extensive neutrophil influx to the site of infection, CD4 T-cells that are recruited to the site of infection will be activated by the cytokines and other inflammatory mediators so as to become optimal targets for HIV infection and replication. Macrophage that are recruited to the site of gonococcal infection may also produce pro-inflammatory cytokines and chemokines to simultaneously activate local CD4 T-cells and recruit further HIV-receptive target cells. Furthermore, the macrophages or DCs can take up the HIV present in the genital microenvironment and travel to distal secondary lymphoid organs where they can further promote HIV infection of CD4 T-cells in these regions.

In considering how anogenital co-infections may influence HIV-1 transmission, it is important to differentiate between effects that alter the *infectiousness* of the infected partner versus those that influence the uninfected partner’s *susceptibility* to infection (Galvin and Cohen, 2004). Given that the HIV-1 titer in genital secretions dictates the likelihood of transmission from an infected person (Quinn et al., 2000), infectiousness will be increased by any factor that increases virus shedding in genital mucus or semen, or perhaps that selects for viral variants that are inherently better able to establish infection. Synergistically in the uninfected partner, susceptibility to infection will be increased by any host-intrinsic factors that provide portals of entry across the mucosal barrier, since this provides access to target CD4+ T cells in the underlying tissues, or that stimulate an immune response that is either ineffective at combatting or, even worse, that promotes HIV-1 replication (Galvin and Cohen, 2004). For example, a co-infecting pathogen may increase an HIV-infected person’s infectiousness by stimulating an immune response that recruits virally infected cells to the genital tract and/or by increasing viral production by HIV-infected cells that are already resident there (Figure 1). Alternatively, mucosal tissue barrier function may be hampered by direct pathogen-induced cytolysis of epithelial cells and/or indirectly by the disruption of epithelial cell junctions in response to inflammatory cytokines elicited by a co-infecting pathogen, increasing an individual’s susceptibility by allowing HIV-1 entry into the underlying tissues (Figure 2).

## HIV-1 Biology

While the intact mucosa typically provides an effective barrier against HIV-1, the virus may normally gain access to underlying target cells via its transcellular transport from the mucosal lumen into the lamina propria through intact epithelial cells, may pass through an non-intact epithelium that has been disrupted by inflammation or by physical stress during sexual activity, or may be taken up by tissue resident dendritic cells (DCs) in the lamina propria as they extend dendrites between epithelial cells to sample the luminal microenvironment for foreign antigens (Tugizov, 2016). Once within the mucosal tissues, activated CD4+ T cells are the primary target for HIV-1 infection. However, other cell types can either directly or indirectly facilitate infection. Most notable in this regard, DCs may express DC-SIGN, a cell surface-expressed receptor that binds the HIV-1 envelope glycoprotein, gp120 (Geijtenbeek et al., 2000). This interaction does not lead to direct DC infection, but the DC may transfer the virus to T cells with which it engages *in trans*, either within the mucosal tissue microenvironment or after the DC migrates into secondary lymphoid organs (Shen et al., 2009). Macrophages can also take up cell-free virus and present it to CD4+ T cells. While macrophages are considered to be intrinsically resistant to HIV-1 infection, they clearly can be infected by HIV-1 since integrated virus is apparent and has been demonstrated within them (Araínga et al., 2017; Clayton et al., 2017). The contribution of these cell types to infection, and the effect that *N. gonorrhoeae* may have on HIV exposure and the outcome of this interaction, will be discussed further below.

Upon contact with CD4+ T cells, the homotrimeric HIV-1 envelope protein (Env) engages cell surface-expressed CD4 and one of its co-receptors, generally either the CXCR4 or CCR5 chemokine receptor, depending on the viral variant’s tropism. This interaction promotes a dramatic conformational change in Env to drive fusion of the HIV-1 and target cell membranes, allowing entry of the viral core into the cytoplasm (Rawle and Harrich, 2018). Once uncoated, the single-stranded RNA genome is reverse transcribed to double-stranded DNA by the pre-formed viral reverse transcriptase; this is a prerequisite for viral genome transport into the nucleus and its integration into the host cell genome. Integration allows establishment of a durable yet transcriptionally silent viral reservoir within long-lived memory T-cells (Finzi et al., 1997; Churchill et al., 2015). However, cellular activation promotes HIV-1 exit from this latent state, driven in part by the T cell receptor-stimulated translocation of active human nuclear factor-kappa B (NF-κB) into the nucleus, where it binds the HIV-1 long terminal repeat (LTR) to initiate transcription of the integrated proviral genome (Mbonye and Karn, 2017). In this context, it is notable that NF-κB is activated by a wide variety of stress responses and danger signals, including by cellular exposure to certain inflammatory cytokines or microbial-associated molecular patterns (MAMPs; also known as pathogen-associated molecular patterns, or PAMPs) (Oeckinghaus et al., 2011; Hayden and Ghosh, 2012). This suggests a variety of mechanisms by which co-infection might promote HIV-1 infection.

## *Neisseria gonorrhoeae* Infection

All STIs have been demonstrated to enhance HIV transmission to some degree, and the epithelial disruption induced by ulcerative STIs such as syphilis provides an obvious mechanism. Among STIs that can increase HIV-1 transmission, however, *N. gonorrhoeae* seems to have a disproportionately large effect on HIV transmission, particularly when considering that it is not an ulcerative infection (Fleming and Wasserheit, 1999; Cohen, 2012). This could be mediated via enhancement of HIV infectiousness in HIV-infected (Figure 1) and/or HIV susceptibility in uninfected individuals (Figure 2). In terms of the former, HIV-1 infected men with gonorrhea were observed to shed ∼10-fold more virus in semen than do men without urethritis, an effect greater than that caused by other non-ulcerative STIs (Cohen et al., 1997). Notably, *N. gonorrhoeae* infection had no impact on viral titers in blood, since these were not different between *N. gonorrhoeae* and HIV-1 co-infected versus HIV-1 alone control groups. However, appropriate and specific antibiotic therapy reduced semen viral shedding in the gonococcal-infected individuals to levels similar to gonorrhea-uninfected controls, suggesting that this was a localized effect that directly correlates with the mucosal gonococcal infection. The global effect of this relationship on HIV-1 spread is compounded by the fact that ∼87 million new gonococcal infections occur each year (Rowley et al., 2019), allowing frequent opportunity for co-exposure in sexually active or at risk individuals.

The gonococcus is an obligately human-restricted pathogen that is highly adapted to colonize mucosal tissues, preferentially the male urethra and female endocervix, but it can also effectively colonize the nasopharynx, rectum and conjunctiva when these tissues become exposed (Britigan et al., 1985; McSheffrey and Gray-Owen, 2015; Unemo et al., 2019). The outcome of gonococcal infection varies, ranging from an absence of any clinical signs to an intensely pathogenic inflammation manifesting as a purulent discharge composed almost entirely of polymorphonuclear neutrophils (Criss and Seifert, 2012). These differences in outcome may depend, in part, on the physiology, tissue structure and innate immune effectors resident within the infected tissues (Edwards and Apicella, 2004; Unemo et al., 2019), since male urethral infections tend to be symptomatic while female endocervical infections are less frequently so; however, asymptomatic infections can occur in men (Handsfield et al., 1974; Kent et al., 2005) and cervicitis does occur in a substantial proportion of women (Unemo et al., 2019). Moreover, gonococcal-associated pathogenesis becomes particularly devastating when the infection ascends into the female endometrium and fallopian tubes, where it can trigger pelvic inflammatory disease (PID) with intense inflammation leading to tissue scarring, chronic pain and infertility (Taylor et al., 2011; Lenz and Dillard, 2018). In humans, gonococcal PID tends to onset with abrupt and intense symptoms, often during the first 10 days after the onset of menses. Sex hormone cycling, which affects both the physiology and microbiome of the female genital tract (Bradley et al., 2018), also impacts the outcome of uterine infection in a mouse model of pelvic inflammatory disease, with a marked increase in gonococcal tissue invasion and inflammation during the progesterone-dependent diestrus stage of the reproductive cycle (Islam et al., 2016). How much these differences are explained by hormonal effects on gonococcal physiology (James and Swanson, 1978; Salit, 1982) versus the physical and immunological integrity of the mucosal barrier (Islam et al., 2016; Lenz and Dillard, 2018) remains unclear.

## *N. gonorrhoeae* Pathogenesis

Studies that have attempted to understand the impact and pathogenesis of *N. gonorrhoeae* in the context of HIV-1 co-infections are inherently challenging because both pathogens are human restricted. Our understanding of how the gonococci contribute to HIV-1 transmission must, therefore, be inferred by combining the insights gained through clinical surveillance of gonococcal and HIV-1 co-infections with those from laboratory-based models developed to study one or the other pathogen. The remainder of this review will bring these results together to develop an integrated model of how *N. gonorrhoeae* impacts HIV-1 shedding and susceptibility to HIV-1 infection, gaining insights from studies exploring the molecular and immunological processes that govern aspects of gonococcal infection with potential to impact upon HIV-1, as well as clinical studies exploring the impact of *N. gonorrhoeae* co-infection on an individual’s susceptibility to HIV-1 or their likelihood to transmit the virus to their partners.

### Cellular Attachment

*Neisseria gonorrhoeae* are not overtly pathogenic in that they do not produce protein-based exotoxins or cause direct host cellular killing, consistent with the fact that infections are often asymptomatic. Pathogenesis is, therefore, a result of neisserial replication within the tissues. To establish infection, the gonococci attach to the epithelia via their type IV pilus, which retracts to allow the bacteria to move through the mucus and establish tight secondary binding between outer membrane protein adhesins on the bacterial surface and their cognate host cellular receptors (Virji, 2009; Lenz and Dillard, 2018). Of these, the gonococcal colony opacity-associated (Opa) proteins have been shown to attach to members of the human carcinoembryonic antigen-related cellular adhesion molecule (CEACAM) family of surface glycoproteins, with the different binding specificities of each Opa protein variant and the varying expression of different CEACAMs on each cell type ultimately determining the outcome of this interaction (Sadarangani et al., 2011; Islam et al., 2018; Yu et al., 2019). Transgenic mouse-based studies have established that Opa protein binding to CEACAM1 and/or CEACAM5 promotes epithelial attachment in the upper and lower female genital tract, respectively (Islam et al., 2018), while Opa binding to the neutrophil-expressed innate decoy receptor CEACAM3 promotes gonococcal engulfment and killing, and elicits a self-propagating pro-inflammatory cytokine response that drives the ongoing recruitment and activation of neutrophils to the infected tissues (Sintsova et al., 2014; Islam et al., 2018). Aside from these direct effects, CEACAM binding also elicits an integrin-dependent increase in epithelial cell affinity for the extracellular matrix, which reduces exfoliation of the infected cell so as to promote infection (Muenzner et al., 2010). The cumulative benefit of these effects presumably explains why bacteria recovered from clinical specimens tend to be expressing one or more of the phase variably-expressed Opa protein variants (James and Swanson, 1978; Swanson et al., 1988; Jerse et al., 1994).

While CEACAM-dependent binding has been the most extensively characterized means of tissue attachment, Opa-independent interactions, including endocervically expressed CR3 binding by the gonococcal pilin-linked glycans (Jennings et al., 2011) and urethrally expressed asialoglycoprotein receptor binding to gonococcal surface glycans (Harvey et al., 2001), can also promote tissue association and affect a cellular response. A key challenge in understanding how the mucosal tissues respond to *N. gonorrhoeae* and, thereby, how co-infection influences HIV transmission, stems from the fact that these and other gonococcal surface ligands are both phase and antigenically variable, allowing stochastic on-off switching of their expression and alteration of the bacteria’s epitope repertoire and phenotype, respectively (Virji, 2009). Thus, a gonococcal population may elicit different effects depending upon which adhesins (and other surface antigens) are expressed, and what cellular receptors are expressed by the infected tissues. One must remain mindful of this when interpreting laboratory-based experiments, but the contribution of this variability to the different outcomes of natural infection must also be considered since each niche will presumably select for the most fit phenotype in an ongoing basis.

### Tissue Response

The specificity of neisserial adhesins for human-derived receptors has led to considerable effort toward modeling molecular aspects of infection using immortalized cell lines, primary cells, and tissues. Classical fallopian tube organ culture studies revealed that gonococci penetrate through non-ciliated epithelia via a transcellular route to emerge into the sub-epithelial spaces (McGee et al., 1983), and caused the sloughing of adjacent (but uninfected) ciliated cells (Melly et al., 1984). More recently, primary human cervical explants have been used to reveal that infection with gonococci that do not express Opa proteins promotes dissociation of intercellular junctions to allow luminal shedding of the infected cells, while epithelia remains intact when the bacteria express CEACAM-specific Opa variants (Wang et al., 2017; Yu et al., 2019), consistent with the effects seen in human CEACAM-expressing transgenic mouse-based studies (Muenzner et al., 2010). When using ureter-derived tissue as a model of male urethral infection, luminally exposed cells that engulf piliated (but non-CEACAM-binding) gonococci were also observed to release their intercellular junctions and be shed, thinning the stratified epithelia and exposing underlying tissues (Mosleh et al., 1997). Considered together, these data suggest that Opa-dependent binding to epithelial-expressed CEACAMs allows attachment and impedes the exfoliation that normally occurs as a defense against bacterial infection, thereby promoting gonococcal infection by maintaining the epithelial integrity. It is notable in this regard that the transitional zone between the ectocervix and endocervix displays very little CEACAM expression and allows gonococcal penetration regardless of their Opa protein expression (Yu et al., 2019), suggesting that this region may be particularly susceptible to gonococcal-induced epithelial thinning to promote exposure to underlying HIV-1 target cells.

Aside from the direct effects of *N. gonorrhoeae* on the epithelium, the inflammatory response to infection can itself interrupt the epithelial barrier. The gonococci have a curious penchant for stimulating inflammation by virtue of their ongoing release of peptidoglycan-derived NOD1 agonists (Lenz et al., 2017), heptose phosphate metabolites that activate the AlpK1-TIFA innate sensor pathway (Gaudet et al., 2015), outer membrane “blebs” containing abundant TLR4 [endotoxin (Pridmore et al., 2003)] and TLR2 [lipoproteins (Fisette et al., 2003), PorB (Zhu et al., 2018)] agonists, and the type IV secretion system-mediated release of TLR9-stimulatory single-stranded chromosomal DNA (Ramsey et al., 2011) into the extracellular milieu. The release of these microbial-associated molecular pattern (MAMP)-containing metabolites is energetically wasteful, implying that the resulting inflammation somehow facilitates gonococcal infection, such as by allowing increased nutrient leakage into the mucosal tissues or by skewing the innate and/or adaptive immune responses so that infection can persist. Regardless, in the context of co-infection with HIV-1, pro-inflammatory cytokines such as TNF are abundantly produced upon gonococcal infection of human fallopian tube cultures, and promote cellular apoptosis and a breach in epithelial integrity (Morales et al., 2006), as well as increased HIV-1 expression in infected CD4+ T cells (Ferreira et al., 2011).

Beyond the effects of *N. gonorrhoeae* on HIV transmission, it is also interesting to consider that HIV-1 may influence the outcome of gonococcal infection. Inflammatory cytokines produced during the acute phase of HIV-1 infection cause a direct loss in epithelial integrity within the intestinal mucosa (Nazli et al., 2010), allowing bacteria and bacterial products to pass into the tissues and cause systemic inflammation that drives further viral replication (Brenchley et al., 2006). Other studies have shown that interaction with HIV-1 envelope protein gp120 may itself disrupt tight and adherens junctions, causing permissiveness to infection by other viral and bacterial pathogens (Nazli et al., 2010; Tugizov, 2016). This would suggest that a synergistic effect may occur, where the inflammatory response to HIV-1 would allow increased gonococcal tissue penetration, which could recruit cellular targets of HIV-1, further disrupt the epithelial barrier, and drive NF-κB-dependent HIV replication. If inflammation and/or tissue penetration are, indeed, of benefit to the gonococci, then this may facilitate infection. However, to our knowledge, there are not yet clinical or epidemiological studies to determine whether chronic HIV-1 infection promotes gonococcal infection or disease.

## Gonococcal-Specific Effects on HIV-1 Target Cell Populations

The hallmark of a gonococcal infection is the pathogenic recruitment of neutrophils to the site of infection, leading to a purulent discharge that results from this prolonged and inappropriately exuberant neutrophil recruitment. Despite this intense inflammatory response, there is curiously little evidence of an adaptive response to gonococcal infection. The answer to this conundrum appears to stem from the Th17-biased response to gonococcal infection, at least in the case of lower genital tract infection in female mice. Specifically, gonococcal infection leads to marked expression of the Th17-related IL-17 and IL-22, without any concomitant appearance of Th1 or Th2 cytokines, or of gonococcal-specific antibodies. When IL-17 signaling was blocked with IL-17-specific antibodies or in IL-17 receptor-deficient mice, neutrophil recruitment decreased and the gonococcal burden increased, implying that IL-17-driven effects combat the infection (Feinen et al., 2010). However, strikingly, the administration of TGFβ-specific antibodies during primary gonococcal infection effectively inhibited the Th17 response (because TGFβ drives Th17 differentiation in mice) but allowed the emergence of a gonococcal-specific adaptive response that protected against re-infection (Liu et al., 2012). A protective memory response could also be elicited by blocking IL-10 signaling (Liu et al., 2014) or by the administration of microencapsulated IL-12 (Liu et al., 2018) during the primary infection. Considered together, these studies suggest that the gonococci have evolved to persist during the Th17-driven inflammation, with the benefit being that this highly polarized response prevents the individual from becoming immune to the infection. What remains unclear is whether this Th17 response is an inherent bias of the female genital tract or whether the gonococci have properties that elicit this outcome, such as through their ongoing and profuse release of MAMPs discussed earlier in this review. Regardless, this effect may directly influence HIV-1 transmission because activated Th17 cells are the primary target for this virus during acute mucosal infection (Gosselin et al., 2010; Prendergast et al., 2010; Alvarez et al., 2013; McKinnon et al., 2015), and may indirectly affect virus susceptibility if HIV-specific adaptive responses were to be inhibited.

### Innate Activation of HIV-Infected CD4 + T Cells

The disparate effects that CD4+ T cells may have on host immune protection, exemplified by the opposing outcomes of Th17 and Th1 effector responses on gonococcal infection discussed above, makes it somewhat foolhardy to try and difficult to extrapolate from cell-based *in vitro* studies to predict the global outcome of infection *in vivo*, particularly considering the central role that these cells play as both a cellular HIV target and drivers of host immunity against HIV infection. Nevertheless, it is reasonable to consider the effect of co-infection on a cellular level. Given that NF-κB-driven transcription is the most dramatic outcome of cellular activation in response to microbial-derived immune agonists, and that this transcription factor governs expression from the HIV-1 LTR, this is an obvious potential link between co-infection and HIV-1 replication. As noted above, pro-inflammatory cytokines expressed by gonococcal-infected genital epithelial cells provoke LTR-driven HIV expression in an NF-κB-dependent manner (Ferreira et al., 2011), revealing an indirect effect that should be shared by any inflammatory stimuli. A more direct effect on HIV-1 replication is apparent with gonococcal-derived TLR2 agonists, which promote HIV-1 uptake and nuclear import of viral DNA in resting primary human CD4+ T cells (Ding et al., 2010). Notable in this regard, gonococcal-derived endotoxin (a TLR4 agonist) does not have a similar effect, consistent with the fact that most other TLRs are not expressed by human CD4+ T cells (Ding et al., 2010; Malott et al., 2013).

Once latent HIV-1 infection has been established, gonococcal infection will elicit a robust viral LTR-dependent transcriptional response. This effect occurs independent of any TLR (or NOD) receptors, and can be elicited by simple exposure of the latently infected CD4+ T cells to bacterial-free culture supernatants from *N. gonorrhoeae*, but does not occur in response to culture supernatants from non-neisserial bacterial species (Chen et al., 2003; Malott et al., 2013). This activity is due to the peculiar propensity for pathogenic and commensal *Neisseria* species to release heptose phosphate-containing metabolites from the ADP-heptose pathway, a biosynthetic cascade that normally leads to the incorporation of heptose into the inner core of gram negative lipopolysaccharides (Malott et al., 2013; Gaudet et al., 2015); other bacteria effectively sequester heptose phosphates within their cytoplasm (Gaudet et al., 2015). These 7-carbon phosphorylated sugars activate innate immune responses in mammalian cells independent of canonical pattern recognition receptor-mediated signaling. Instead, they are directly bound by the cytosolic alpha kinase 1 (AlpK1). Heptose phosphate binding activates the kinase so that it can phosphorylate TIFA, which then self-associates into large cytosolic TIFAsome structures that function as a scaffold to drive NF-κB-mediated transcription (Gaudet et al., 2015; Milivojevic et al., 2017; Adekoya et al., 2018; Zhou et al., 2018). Heptose phosphates therefore provide a direct link between gonococcal infection and HIV-1 replication in CD4+ T cells by the virtue of NF-κB activation through TIFA-AlpK1 axis.

### CEACAM1-Mediated Suppression of T Cell Responses

While innate drivers of inflammation stimulate HIV-1 transcription, gonococcal Opa protein binding to human CEACAM1 can broadly suppress CD4+ T cell responses to T cell receptor engagement and certain other activating stimuli (Boulton and Gray-Owen, 2002). Very little CEACAM1 is apparent on resting lymphocytes, but its expression is upregulated upon cellular activation. Unlike other immune cells, CD4+ T-cells are unable to engulf gonococci that attach to their surface, leading to a stable association between Opa protein-expressing gonococci and CEACAM1 on the cell surface (Lee et al., 2008). When the T cell receptor becomes engaged by antigen presenting cells, it would normally unleash a kinase-dependent signaling cascade that activates the T cell. However, when CEACAM1 is engaged by gonococci (Lee et al., 2008) or gonococcal-derived outer membrane blebs (Lee et al., 2007), tyrosine residues within the immune receptor tyrosine-based inhibitory motif (ITIM) in the CEACAM1 cytoplasmic domain also become phosphorylated, creating docking sites to recruit SHP-1 and SHP-2 phosphatases (Boulton and Gray-Owen, 2002; Gray-Owen and Blumberg, 2006; Lee et al., 2008). Once activated, these enzymes rapidly dephosphorylate the T cell receptor and downstream signaling effectors, shutting down the activating response (Boulton and Gray-Owen, 2002; Lee et al., 2007). This immune inhibitory effect is compounded by the fact that Opa protein binding to CEACAM1 on DCs reprograms their maturation such that they are not able to effectively present antigen to T cells (Yu et al., 2013). In the context of HIV-1 co-infection, the hampering of T cell responses may have two different effects. First, given that the nuclear localization of NF-κB is a primary outcome of T cell receptor engagement, it seems reasonable to assume that the phosphatase activation will increase the threshold of cellular activation required to drive HIV-1 from latency. Second, the central role that CD4+ T cells play in the adaptive immune response suggests that CEACAM1 binding may suppress development of an adaptive response to the infection. While the reduced inflammation and absence of memory response may both facilitate gonococcal infection, their cumulative effect on HIV-1 infection and immunity are difficult to test given that gonococcal Opa proteins only bind human (and not mouse or other) CEACAM1.

### Gonococcal Interaction With Antigen Presenting Cells

Macrophage and DCs reside as sentinels within the submucosa to detect microbes infiltrating the otherwise sterile tissues, and then direct the immune responses to eliminate the invading pathogens. After capture of microbial-derived products, they migrate to secondary lymphoid organs to present foreign epitopes to T cells and thereby elicit a pathogen-specific adaptive response. Both phagocyte types express CD4 and the HIV-1 co-receptors, CXCR4 and CCR5, which facilitate viral uptake (Martin-Moreno and Munoz-Fernandez, 2019). However, while macrophages may be productively infected in certain contexts, DCs express innate viral restriction factors so that they rarely become infected, and produce relatively little virus when they do (Smed-Sorensen et al., 2005). Despite this fact, DCs are key to the establishment of infection, in part because they can project dendrites between epithelial cells and come in contact with virus as they sample the mucosal lumen, facilitating viral transport across an otherwise intact epithelial barrier (Martin-Moreno and Munoz-Fernandez, 2019). Further exacerbating this effect, HIV-1 binding to DC-SIGN facilitates its retention on the dendritic cell surface (Geijtenbeek et al., 2000), allowing the virus to be carried to the lymph nodes and then transferred to CD4+ T cells as they engage with the phagocyte (Martin-Moreno and Munoz-Fernandez, 2019). Events that promote macrophage or dendritic cell sampling and/or movement to the lymph nodes, as might occur during gonococcal co-infection, would presumably facilitate this process.

While tissue resident DCs may resist infection, there is some indication that exposure to certain stimuli might affect this outcome. For instance, *in vitro* studies indicate that gonococcal co-infection of monocyte-derived DCs increases their likelihood for HIV-1 infection based upon the emergence of virally derived reverse transcriptase activity, and this effect can be reproduced through the administration of purified gonococcal PorB or lipoproteins, both of which are TLR2 agonist, or peptidoglycan (Zhang et al., 2005). This study did not correlate cellular phenotype with viral expression on a single cell level, and did not quantify the proportion of cells that became infected or number of viral particles produced by the human cells, so how productive the infections were and whether the virus-expressing cells were actually DCs remains unclear. Notably, a separate study confirmed that TLR2 agonists enhanced HIV-1 production from human DCs, but also observed that TLR4 agonists instead suppressed HIV expression by virtue of their distinctive ability to stimulate production of type-I interferons, which upregulate cell autonomous immune defenses (Thibault et al., 2009). Given that this study used purified agonists, it remains unknown how the combination of these and other bacterial-derived innate agonists might combine to influence HIV-1 replication *in vivo*.

Distinct from the broad involvement of conventional DCs in coordinating the adaptive response to a broad range of microbes, plasmacytoid dendritic cells (pDCs) are generally considered to be specialized for antiviral immunity (Martin-Moreno and Munoz-Fernandez, 2019). In this context, it is noteworthy that the gonococci unexpectedly elicit a potent pDC response, leading to a robust expression of interferon-α (IFNα), sufficient to cause a profound inhibition of HIV-1 replication in *ex vivo* cultures of peripheral blood cells from HIV-infected patients (Dobson-Belaire et al., 2010). Curiously, this effect is a consequence of the tendency of gonococci to liberate their genomic DNA, either by cellular lysis or their type 4 secretion system, which is detected by TLR9. At first glance, this would seem to contradict the HIV-1 stimulatory effect that has been ascribed to *N. gonorrhoeae*, however pDCs are a double-edged sword with respect to HIV-1 because the IFNα response can independently upregulate expression of viral-specific cellular defenses and recruit activated immune cells that can be infected by the virus (Martin-Moreno and Munoz-Fernandez, 2019). The consequences of this effect remain difficult to surmise without purposeful mucosal sampling of co-infected patients.

In contrast to DCs, tissue resident macrophages are an important latent reservoir of HIV-1 (Meltzer et al., 1990; Clayton et al., 2017). While macrophages are typically infected following CD4 and CCR5 co-receptor engagement, CD4+ T cells remain the preferred HIV-1 target because macrophage display much lower CD4 on their surface. Viral variants that effectively target macrophage have evolved Env protein variants that bind CD4 with much higher affinity, which compensates for its low surface density (Arrildt et al., 2015). A recent study observed that macrophages resident within the human penile urethra can harbor integrated HIV-1, and that these produce infectious virions upon exposure to the TLR4 agonist lipopolysaccharide (Ganor et al., 2019). In the context of male urethral infection by *N. gonorrhoeae*, viral shedding could then be stimulated either directly by macrophage exposure to the gonococci or their liberated products, or indirectly through the exuberant inflammatory cytokine response that is typical of gonorrhea. While yet undescribed, it seems reasonable to assume that a similar effect could occur within the female genital tract.

Beyond the stable effects of *N. gonorrhoeae* on viral replication, it is important to consider that ongoing phase variation might also cause different effects depending upon the virulence factors expressed. Notable in this regard, lipooligosaccharide (LOS) surface structures displaying terminal *N-*acetylglucosamine or *N-*acetylgalactosamine residues are detected by the C-type lectins DC-SIGN on DCs and by MGL on macrophage, respectively. These immune modulatory receptors have the potential to alter the phagocytic responses to other activating stimuli. Consistent with this, gonococcal isolates expressing different LOS structures were found to elicit modest but significant differences in the pattern of cytokines expressed upon *in vitro* infection of these phagocytes, leading to speculation that the adaptive response may skew toward a Th1, Th2, or Th17 bias depending on what phase variable glycan structures are present (van Vliet et al., 2009). As discussed above, phase variable Opa protein-dependent binding to CEACAM1 expressed by DCs can also effectively suppress their maturation in response to activating stimuli including gonococcal infection, preventing the cells from stimulating both CD4+ T cell and CD8+ T cell responses (Yu et al., 2013). It is enticing to think that these effects may have contributed to the observed Th17 immunological bias that suppresses a protective memory response in the mouse lower genital tract infection studies described above. However, whether these differences actually influence the global immune response to *N. gonorrhoeae* and/or HIV-1 during mucosal infection remains to be explored.

### The Confounding Effect of Antimicrobial Peptides

It seems intuitive that HIV-1 may benefit from the gonococcal effects aimed at actively suppressing or misguiding otherwise effective immune defenses, or from stimulating an inflammatory response that drives viral expression. However, *N. gonorrhoeae* also triggers the release of neutrophil and epithelial-derived antimicrobial peptides during female genital infection (Wiesenfeld et al., 2002) and gonococcal urethritis in men (Porter et al., 2005). In fact, increased recovery of neutrophil-derived peptides in vaginal swabs is a highly sensitive marker for endometritis among women infected with *N. gonorrhoeae* (Wiesenfeld et al., 2002). While the gonococci can resist most cationic peptides through a combination of lipid A modification and/or efflux pump expression (Kandler et al., 2016), their effect on HIV-1 susceptibility *in vivo* is not so clear. For example, while HIV-1 tends to be susceptible to many antimicrobial peptides *in vitro*, either directly or via activation of innate cellular responses (Klotman and Chang, 2006; Furci et al., 2007), but α-defensins 5 and 6 have been shown to enhance the infectivity of CCR5-tropic HIV-1 by promoting viral aggregation and attachment to the target cell surface (Klotman et al., 2008; Rapista et al., 2011). Notably, many antimicrobial peptides are secreted in a propeptide form that must be processed by neutrophil-released proteases (Porter et al., 2005). Therefore, the relative expression of different peptides and local density of neutrophils, which is obviously high during gonococcal-induced inflammation, is a delicate balance that in theory could combine to either facilitate or inhibit HIV-1 infection. Unfortunately, it seems that the balance is tilted toward increased HIV susceptibility: despite the fact that increased genital levels of α-defensins and cathelicidins (LL-37) were associated with an increased ability of genital secretions to neutralize HIV *ex vivo*, these increases were also associated with increased subsequent HIV acquisition in both women (Levinson et al., 2009) and men (Hirbod et al., 2014).

## Gonococcal Effects on the HIV-1 Specific Adaptive Response

In women, cervicitis is evident as a purulent discharge consisting almost entirely of neutrophils. While the numbers are smaller, it is notable that there is a significant increase in endocervical CD4+, CD8+, and γδ T cells during uncomplicated gonococcal infection regardless of whether cervicitis is apparent or not (Levine et al., 1998). These effects are a highly localized in that there is no difference in the number of leukocytes in ectocervical or vaginal specimens taken from women with or without *N. gonorrhoeae* infection (Levine et al., 1998), and they do not result from a global increase in systemic lymphocyte counts, which don’t significantly change during gonococcal infection (Kaul et al., 2002). It remains unclear whether the apparently targeted recruitment of leukocytes reflects differences in gonococcal attachment or penetration through the epithelial barrier at the endocervix, or whether there are distinct inflammatory responses by epithelia along the genital tract. Regardless, considering that the endocervix is considered to be the prime site for infection with HIV-1, this suggests an obvious site for direct and indirect interactions to occur.

Despite the localized nature of uncomplicated gonococcal infection, there are systemic effects of gonorrhea. Most notably, HIV-1 viremia increases during gonococcal cervicitis (Anzala et al., 2000). While the number of T cells does not change in blood, functional studies suggest that a smaller proportion of HIV epitope-specific CD8+ T cells express IFNγ during gonococcal infection than do after the bacterial infection is treated (Kaul et al., 2002). This appears to be a global effect as a similar reduction in responses to CMV epitopes was also apparent (Kaul et al., 2002), and may reflect a systemic skewing of the T cell response rather than a global suppression of immunity (Anzala et al., 2000). The effect of gonococcal infection is transient in that any effects on lymphocyte response, systemic HIV-1 load or localized viral shedding abate once the bacterial infection is cleared.

Given the chronic nature of HIV-1 infection, clinical studies understandably tend to explore the interactions between *N. gonorrhoeae* and the virus by monitoring changes (or lack thereof) when HIV-infected individuals acquire a gonococcal infection. A rare opportunity to describe the effects of gonococcal infection on HIV-specific immune responses occurred during a large HIV-1 prevention trial involving high risk HIV-negative female sex workers in Nairobi, Kenya, since it involved monthly screening for STIs, including gonorrhea (Sheung et al., 2008). Thirty five of 466 participants acquired HIV-1 during the course of this trial. Quite unexpectedly, the intensity and breadth of HIV-specific CD8 T-cell responses were significantly higher in female sex workers who acquired HIV-1 during a period in which they had a gonococcal infection relative to that apparent in women who were not co-infected (Sheung et al., 2008). This difference was not simply due to increased viral shedding by the transmitting male partner, since *Chlamydia trachomatis* and *Trichomonas vaginalis* also cause urethritis in men but coincident infection with these did not have a similar immune-enhancing effect. Given that gonococcal co-infection did not appear to influence the viral load setpoint once infection is established (Sheung et al., 2008), and that individuals within this cohort have a 5-fold increased risk of acquiring HIV-1 (Kaul et al., 2004), the increased CD8 response was not protective. Instead, it seems reasonable to consider that the increased adaptive response may stem from a combination of higher exposure from the gonococcal-infected seminal fluid and an increased susceptibility of the female partner, perhaps due to the gonococcal-dependent recruitment of target cells into the genital tract or disruption of the epithelial barrier, leading to a higher acute exposure to the virus. In this case, the increased susceptibility and increased immune response may effectively offset each other.

### Modeling the Mucosal Response

The relationship between *N. gonorrhoeae* and HIV-1 are complex, and the observational nature of clinical studies prevents the assignment of causality to any effects seen. Models intended to understand molecular and immunologic interactions between the pathogens are difficult to establish, since both *N. gonorrhoeae* and HIV-1 are human-restricted pathogens. Indeed, gorilla, orangutan and chimpanzees may be the only hosts that these two pathogens might naturally co-infect (McGee et al., 1990; Gray-Owen and Schryvers, 1993), and these are neither financially or (more importantly), ethically reasonable models. However, the recent advent of highly elegant mouse models for the study of HIV-1 provides an opportunity to explore some aspects of co-infection. In particular, the engraftment of human CD34^+^ fetal liver or umbilical cord-derived blood stem cells into highly immunodeficient NSG (NOD/*Ltsz-scid*/*scid*γ*c*^*null*^) mice allows reconstitution of a leukocyte pool that is human-derived, allowing chronic HIV-1 infection of the human CD4+ T cells (Denton and Garcia, 2011). When these mice were vaginally challenged with *N. gonorrhoeae*, they displayed increased shedding of HIV-1 in genital secretions (Xu et al., 2018). This response occurred without a concomitant increase in systemic viremia, suggesting that the gonococcal infection was stimulating local HIV-1 production within cells of the genital tract. While this model is not trivial to establish, it provides a tractable system to gain mechanistic insight and explore novel therapeutic interventions.

## Conclusion

Sexually transmitted infections have been referred to as a hidden epidemic (Institute of Medicine, Committee on Prevention and Control of Sexually Transmitted Diseases, 1997; Cohen, 2012), both because they are not openly discussed due to stigma, and also because they may be asymptomatic in over 80% of cases and so are infrequently recognized as an STI since a prospective study of women who are at high risk of HIV acquisition found that only 12.3% of women infected with a pathogen known to cause vaginal discharge had any signs or symptoms of infection (Mlisana et al., 2012). Thus, even though these co-infections are currently treatable, they continue to drive HIV transmission while they smolder undetected due to stigma and under-diagnosis. This situation is exacerbated in the case of *N. gonorrhoeae* since the recent emergence of multi-drug resistant isolates may soon reduce our ability to effectively treat the infection (Wi et al., 2017). In the absence of an HIV vaccine on the horizon, it is reasonable to consider that interventions targeting these co-pathogens and other pathogens that facilitate HIV transmission may represent one of the achievable short to medium-term strategies to reduce the spread of HIV-1. Considering the substantial impact that gonorrhea in particular has on HIV transmission within a population, further studies aimed to understand the molecular and immunologic determinants of this interaction will hopefully reveal new prevention avenues. A vaccine that confers sterilizing immunity against *N. gonorrhoeae* is a particularly enticing goal when considering that this bacterium does not live outside of humans, which suggests that it could be eradicated. Despite political and economic barriers to the implementation of interventions against STIs will always be contentious, the potential for a lasting positive global health impact on two major pandemics means that there is no better time than the present to get this work underway.

## Author Contributions

All authors listed have made a substantial, direct and intellectual contribution to the work, and approved it for publication.

## Conflict of Interest

The authors declare that the research was conducted in the absence of any commercial or financial relationships that could be construed as a potential conflict of interest.

## References

[B1] Adekoya I. A. Guo C. X. Gray-Owen S. D. Cox A. D. Sauvageau J. (2018). d-Glycero-beta-d-manno-heptose 1-phosphate and d-Glycero-beta-d-manno-heptose 1,7-biphosphate are both innate immune agonists. *J. Immunol.* 201 2385–2391. 10.4049/jimmunol.1801012 30224513

[B2] Alvarez Y. Tuen M. Shen G. Nawaz F. Arthos J. Wolff M. J. (2013). Preferential HIV infection of CCR6+ Th17 cells is associated with higher levels of virus receptor expression and lack of CCR5 ligands. *J. Virol.* 87 10843–10854. 10.1128/JVI.01838-13 23903844 PMC3807416

[B3] Anzala A. O. Simonsen J. N. Kimani J. Ball T. B. Nagelkerke N. J. Rutherford J. (2000). Acute sexually transmitted infections increase human immunodeficiency virus type 1 Plasma Viremia. increase plasma type 2 Cytokines, and decrease CD4 cell counts. *J. Infect. Dis.* 182 459–466. 10.1086/315733 10915076

[B4] Araínga M. Edagwa B. Mosley R. L. Poluektova L. Y. Gorantla S. Gendelman H. E. (2017). A mature macrophage is a principal HIV-1 cellular reservoir in humanized mice after treatment with long acting antiretroviral therapy. *Retrovirology* 14:17. 10.1186/s12977-017-0344-7 28279181 PMC5345240

[B5] Arrildt K. T. LaBranche C. C. Joseph S. B. Dukhovlinova E. N. Graham W. D. Ping L. H. (2015). Phenotypic correlates of HIV-1 macrophage tropism. *J. Virol.* 89 11294–11311. 10.1128/JVI.00946-15 26339058 PMC4645658

[B6] Arts E. J. Hazuda D. J. (2012). HIV-1 antiretroviral drug therapy. *Cold Spring Harb. Perspect. Med.* 2:a007161. 10.1101/cshperspect.a007161 22474613 PMC3312400

[B7] Atashili J. Poole C. Ndumbe P. M. Adimora A. A. Smith J. S. (2008). Bacterial vaginosis and HIV acquisition: a meta-analysis of published studies. *AIDS* 22 1493–1501. 10.1097/QAD.0b013e3283021a37 18614873 PMC2788489

[B8] Bain L. E. Nkoke C. Noubiap J. J. N. (2017). UNAIDS 90–90–90 targets to end the AIDS epidemic by 2020 are not realistic: comment on “Can the UNAIDS 90–90–90 target be achieved? A systematic analysis of national HIV treatment cascades”. *BMJ Glob. Health* 2 e000227–e000227. 10.1136/bmjgh-2016-000227 28589026 PMC5435269

[B9] Boulton I. C. Gray-Owen S. D. (2002). Neisserial binding to CEACAM1 arrests the activation and proliferation of CD4+ T lymphocytes. *Nat. Immunol.* 3 229–236. 10.1038/ni769 11850628

[B10] Bradley F. Birse K. Hasselrot K. Noel-Romas L. Introini A. Wefer H. (2018). The vaginal microbiome amplifies sex hormone-associated cyclic changes in cervicovaginal inflammation and epithelial barrier disruption. *Am. J. Reprod. Immunol.* 80:e12863. 10.1111/aji.12863 29709092

[B11] Brenchley J. M. Price D. A. Schacker T. W. Asher T. E. Silvestri G. Rao S. (2006). Microbial translocation is a cause of systemic immune activation in chronic HIV infection. *Nat. Med.* 12 1365–1371. 10.1038/nm1511 17115046

[B12] Britigan B. E. Cohen M. S. Sparling P. F. (1985). Gonococcal infection: a model of molecular pathogenesis. *N. Engl. J. Med.* 312 1683–1694. 10.1056/NEJM198506273122606 2860565

[B13] Chen A. Boulton I. C. Pongoski J. Cochrane A. Gray-Owen S. D. (2003). Induction of HIV-1 long terminal repeat-mediated transcription by *Neisseria gonorrhoeae*. *AIDS* 17 625–628. 10.1097/01.aids.0000050840.06065.35 12598784

[B14] Churchill M. J. Deeks S. G. Margolis D. M. Siliciano R. F. Swanstrom R. (2015). HIV reservoirs: what, where and how to target them. *Nat. Rev. Microbiol.* 14 55–60. 10.1038/nrmicro.2015.5 26616417

[B15] Clayton K. L. Garcia V. Clements J. E. Walker B. D. (2017). HIV infection of macrophages: implications for pathogenesis and cure. *Pathog. Immun.* 2:179. 10.20411/pai.v2i2.204 28752134 PMC5526341

[B16] Cohen M. S. (2012). Classical sexually transmitted diseases drive the spread of HIV-1: back to the future. *J. Infect. Dis.* 206 1–2. 10.1093/infdis/jis303 22517911

[B17] Cohen M. S. Chen Y. Q. McCauley M. Gamble T. Hosseinipour M. C. Kumarasamy N. (2016). Antiretroviral Therapy for the Prevention of HIV-1 Transmission. *N. Engl. J. Med.* 375 830–839. 10.1056/NEJMoa1600693 27424812 PMC5049503

[B18] Cohen M. S. Hoffman I. F. Royce R. A. Kazembe P. Dyer J. R. Daly C. C. (1997). Reduction of concentration of HIV-1 in semen after treatment of urethritis: implications for prevention of sexual transmission of HIV-1. AIDSCAP Malawi Research Group. *Lancet* 349 1868–1873. 10.1016/s0140-6736(97)02190-9 9217758

[B19] Criss A. K. Seifert H. S. (2012). A bacterial siren song: intimate interactions between *Neisseria* and neutrophils. *Nat. Rev. Microbiol.* 10 178–190. 10.1038/nrmicro2713 22290508 PMC3569855

[B20] Cuevas J. M. Geller R. Garijo R. Lopez-Aldeguer J. Sanjuan R. (2015). Extremely High Mutation Rate of HIV-1 In Vivo. *PLoS Biol.* 13:e1002251. 10.1371/journal.pbio.1002251 26375597 PMC4574155

[B21] Denton P. W. Garcia J. V. (2011). Humanized mouse models of HIV infection. *AIDS Rev.* 13 135–148. 21799532 PMC3741405

[B22] Ding J. Rapista A. Teleshova N. Mosoyan G. Jarvis G. A. Klotman M. E. (2010). *Neisseria gonorrhoeae* enhances HIV-1 infection of primary resting CD4+ T cells through TLR2 activation. *J. Immunol.* 184 2814–2824. 10.4049/jimmunol.0902125 20147631 PMC3739425

[B23] Dobson-Belaire W. N. Rebbapragada A. Malott R. J. Yue F. Y. Kovacs C. Kaul R. (2010). *Neisseria gonorrhoeae* effectively blocks HIV-1 replication by eliciting a potent TLR9-dependent interferon-α response from plasmacytoid dendritic cells. *Cell. Microbiol.* 12 1703–1717. 10.1111/j.1462-5822.2010.01502.x 20735437

[B24] Edwards J. L. Apicella M. A. (2004). The molecular mechanisms used by *Neisseria gonorrhoeae* to initiate infection differ between men and women. *Clin. Microbiol. Rev.* 17 965–981. 10.1128/CMR.17.4.965-981.2004 15489357 PMC523569

[B25] Feinen B. Jerse A. E. Gaffen S. L. Russell M. W. (2010). Critical role of Th17 responses in a murine model of *Neisseria gonorrhoeae* genital infection. *Mucosal Immunol.* 3:312. 10.1038/mi.2009.139 20107432 PMC2857675

[B26] Ferreira V. H. Kafka J. K. Kaushic C. (2014). Influence of common mucosal co-factors on HIV infection in the female genital tract. *Am. J. Reprod. Immunol.* 71 543–554. 10.1111/aji.12221 24617528

[B27] Ferreira V. H. Nazli A. Khan G. Mian M. F. Ashkar A. A. Gray-Owen S. (2011). Endometrial epithelial cell responses to coinfecting viral and bacterial pathogens in the genital tract can activate the HIV-1 LTR in an NF{kappa}B-and AP-1-dependent manner. *J. Infect. Dis.* 204 299–308. 10.1093/infdis/jir260 21673042

[B28] Finzi D. Hermankova M. Pierson T. Carruth L. M. Buck C. Chaisson R. E. (1997). Identification of a reservoir for HIV-1 in patients on highly active antiretroviral therapy. *Science* 278 1295–1300. 10.1126/science.278.5341.1295 9360927

[B29] Fisette P. L. Ram S. Andersen J. M. Guo W. Ingalls R. R. (2003). The Lip lipoprotein from *Neisseria gonorrhoeae* stimulates cytokine release and NF-kappaB activation in epithelial cells in a Toll-like receptor 2-dependent manner. *J. Biol. Chem.* 278 46252–46260. 10.1074/jbc.M306587200 12966099

[B30] Fleming D. T. Wasserheit J. N. (1999). From epidemiological synergy to public health policy and practice: the contribution of other sexually transmitted diseases to sexual transmission of HIV infection. *Sex Transm. Infect.* 75 3–17. 10.1136/sti.75.1.3 10448335 PMC1758168

[B31] Furci L. Sironi F. Tolazzi M. Vassena L. Lusso P. Lindbom L. (2007). Alpha-defensins block the early steps of HIV-1 infection: interference with the binding of gp120 to CD4. *Blood* 109 2928–2935. 10.1182/blood-2006-05-024489 17132727

[B32] Galvin S. R. Cohen M. S. (2004). The role of sexually transmitted diseases in HIV transmission. *Nat. Rev. Microbiol.* 2 33–42. 10.1038/nrmicro794 15035007

[B33] Ganor Y. Real F. Sennepin A. Dutertre C.-A. Prevedel L. Xu L. (2019). HIV-1 reservoirs in urethral macrophages of patients under suppressive antiretroviral therapy. *Nat. Microbiol.* 4 633–644. 10.1038/s41564-018-0335-z 30718846

[B34] Gaudet R. G. Sintsova A. Buckwalter C. M. Leung N. Cochrane A. Li J. (2015). INNATE IMMUNITY. Cytosolic detection of the bacterial metabolite HBP activates TIFA-dependent innate immunity. *Science* 348 1251–1255. 10.1126/science.aaa4921 26068852

[B35] Geijtenbeek T. B. H. Kwon D. S. Torensma R. van Vliet S. J. van Duijnhoven G. C. F. Middel J. (2000). DC-SIGN, a dendritic cell–specific HIV-1-binding protein that enhances trans-Infection of T Cells. *Cell* 100 587–597. 10.1016/S0092-8674(00)80694-7 10721995

[B36] Gosselin A. Monteiro P. Chomont N. Diaz-Griffero F. Said E. A. Fonseca S. (2010). Peripheral blood CCR4+CCR6+ and CXCR3+CCR6+CD4+ T cells are highly permissive to HIV-1 infection. *J. Immunol.* 184 1604–1616. 10.4049/jimmunol.0903058 20042588 PMC4321756

[B37] Gray-Owen S. D. Blumberg R. S. (2006). CEACAM1: contact-dependent control of immunity. *Nat. Rev. Immunol.* 6 433–446. 10.1038/nri1864 16724098

[B38] Gray-Owen S. D. Schryvers A. B. (1993). The interaction of primate transferrins with receptors on bacteria pathogenic to humans. *Microb. Pathog.* 14 389–398. 10.1006/mpat.1993.1038 8366816

[B39] Handsfield H. H. Lipman T. O. Harnisch J. P. Tronca E. Holmes K. K. (1974). Asymptomatic gonorrhea in men. Diagnosis, natural course, prevalence and significance. *N. Engl. J. Med.* 290 117–123. 10.1056/NEJM197401172900301 4202519

[B40] Harvey H. A. Jennings M. P. Campbell C. A. Williams R. Apicella M. A. (2001). Receptor-mediated endocytosis of *Neisseria gonorrhoeae* into primary human urethral epithelial cells: the role of the asialoglycoprotein receptor. *Mol. Microbiol.* 42 659–672. 10.1046/j.1365-2958.2001.02666.x 11722733

[B41] Hayden M. S. Ghosh S. (2012). NF-kappaB, the first quarter-century: remarkable progress and outstanding questions. *Genes Dev.* 26 203–234. 10.1101/gad.183434.111 22302935 PMC3278889

[B42] Hirbod T. Kong X. Kigozi G. Ndyanabo A. Serwadda D. Prodger J. L. (2014). HIV acquisition is associated with increased antimicrobial peptides and reduced HIV neutralizing IgA in the foreskin prepuce of uncircumcised men. *PLoS Pathog.* 10:e1004416. 10.1371/journal.ppat.1004416 25275513 PMC4183701

[B43] Institute of Medicine, Committee on Prevention and Control of Sexually Transmitted Diseases (1997). *The Hidden Epidemic: Confronting Sexually Transmitted Diseases*, eds Eng T. R. Butler W. T. Washington, DC: Institute of Medicine, Committee on Prevention and Control of Sexually Transmitted Diseases.

[B44] Islam E. A. Anipindi V. C. Francis I. Shaik-Dasthagirisaheb Y. Xu S. Leung N. (2018). Specific binding to differentially-expressed human CEACAMs determines the outcome of *Neisseria gonorrhoeae* infections along the female reproductive tract. *Infect. Immun.* 86:e00092-18. 10.1128/IAI.00092-18 29760215 PMC6056862

[B45] Islam E. A. Shaik-Dasthagirisaheb Y. Kaushic C. Wetzler L. M. Gray-Owen S. D. (2016). The reproductive cycle is a pathogenic determinant during gonococcal pelvic inflammatory disease in mice. *Mucosal Immunol.* 9 1051–1064. 10.1038/mi.2015.122 26693700 PMC4915993

[B46] James J. F. Swanson J. (1978). Studies on gonococcus infection. XIII. Occurrence of color/opacity colonial variants in clinical cultures. *Infect. Immun.* 19 332–340. 10.1128/iai.19.1.332-340.1978 415007 PMC414084

[B47] Jennings M. P. Jen F. E. Roddam L. F. Apicella M. A. Edwards J. L. (2011). *Neisseria gonorrhoeae* pilin glycan contributes to CR3 activation during challenge of primary cervical epithelial cells. *Cell Microbiol.* 13 885–896. 10.1111/j.1462-5822.2011.01586.x 21371235 PMC3889163

[B48] Jerse A. E. Cohen M. S. Drown P. M. Whicker L. G. Isbey S. F. Seifert H. S. (1994). Multiple gonococcal opacity proteins are expressed during experimental urethral infection in the male. *J. Exp. Med.* 179 911–920. 10.1084/jem.179.3.911 8113683 PMC2191399

[B49] Kandler J. L. Holley C. L. Reimche J. L. Dhulipala V. Balthazar J. T. Muszynski A. (2016). The MisR response regulator Is necessary for intrinsic cationic antimicrobial peptide and aminoglycoside resistance in *Neisseria gonorrhoeae*. *Antimicrob. Agents Chemother.* 60 4690–4700. 10.1128/AAC.00823-16 27216061 PMC4958169

[B50] Kaul R. Kimani J. Nagelkerke N. J. Fonck K. Ngugi E. N. Keli F. (2004). Monthly antibiotic chemoprophylaxis and incidence of sexually transmitted infections and HIV-1 infection in Kenyan sex workers: a randomized controlled trial. *JAMA* 291 2555–2562. 10.1001/jama.291.21.2555 15173146

[B51] Kaul R. Pettengell C. Sheth P. M. Sunderji S. Biringer A. MacDonald K. (2008). The genital tract immune milieu: an important determinant of HIV susceptibility and secondary transmission. *J. Reprod. Immunol.* 77 32–40. 10.1016/j.jri.2007.02.002 17395270

[B52] Kaul R. Saa L. Gillespie G. Kimani J. Dong T. Kiama P. (2002). Gonococcal cervicitis is associated with reduced systemic CD8+ T cell responses in human immunodeficiency virus type 1-infected and exposed, uninfected sex workers. *J. Infect. Dis.* 185 1525–1529. 10.1086/340214 11992292

[B53] Kent C. K. Chaw J. K. Wong W. Liska S. Gibson S. Hubbard G. (2005). Prevalence of rectal, urethral, and pharyngeal chlamydia and gonorrhea detected in 2 clinical settings among men who have sex with men: san Francisco. California, 2003. *Clin. Infect. Dis.* 41 67–74. 10.1086/430704 15937765

[B54] Klotman M. E. Chang T. L. (2006). Defensins in innate antiviral immunity. *Nat. Rev. Immunol.* 6 447–456. 10.1038/nri1860 16724099

[B55] Klotman M. E. Rapista A. Teleshova N. Micsenyi A. Jarvis G. A. Lu W. (2008). *Neisseria gonorrhoeae*-induced human defensins 5 and 6 increase HIV infectivity: role in enhanced transmission. *J. Immunol.* 180 6176–6185. 10.4049/JIMMUNOL.180.9.6176 18424739 PMC3042429

[B56] Langford S. E. Ananworanich J. Cooper D. A. (2007). Predictors of disease progression in HIV infection: a review. *AIDS Res. Ther.* 4:11. 10.1186/1742-6405-4-11 17502001 PMC1887539

[B57] Lee H. S. Boulton I. C. Reddin K. Wong H. Halliwell D. Mandelboim O. (2007). Neisserial outer membrane vesicles bind the coinhibitory receptor carcinoembryonic antigen-related cellular adhesion molecule 1 and suppress CD4+ T lymphocyte function. *Infect. Immun.* 75 4449–4455. 10.1128/IAI.00222-07 17620353 PMC1951172

[B58] Lee H. S. Ostrowski M. A. Gray-Owen S. D. (2008). CEACAM1 dynamics during *Neisseria gonorrhoeae* suppression of CD4+ T lymphocyte activation. *J. Immunol.* 180 6827–6835. 10.4049/jimmunol.180.10.6827 18453603

[B59] Lenz J. D. Dillard J. P. (2018). Pathogenesis of *Neisseria gonorrhoeae* and the host defense in ascending infections of human fallopian tube. *Front. Immunol.* 9:2710. 10.3389/fimmu.2018.02710 30524442 PMC6258741

[B60] Lenz J. D. Hackett K. T. Dillard J. P. (2017). A single dual-function enzyme controls the production of inflammatory NOD agonist peptidoglycan fragments by *Neisseria gonorrhoeae*. *mBio* 8:e01464-17. 10.1128/mBio.01464-17 29042497 PMC5646250

[B61] Levi J. Raymond A. Pozniak A. Vernazza P. Kohler P. Hill A. (2016). Can the UNAIDS 90-90-90 target be achieved? A systematic analysis of national HIV treatment cascades. *BMJ Glob. Health* 1:e000010. 10.1136/bmjgh-2015-000010 28588933 PMC5321333

[B62] Levine W. C. Pope V. Bhoomkar A. Tambe P. Lewis J. S. Zaidi A. A. (1998). Increase in endocervical CD4 lymphocytes among women with nonulcerative sexually transmitted diseases. *J. Infect. Dis.* 177 167–174. 10.1086/513820 9419184

[B63] Levinson P. Kaul R. Kimani J. Ngugi E. Moses S. MacDonald K. S. (2009). Levels of innate immune factors in genital fluids: association of alpha defensins and LL-37 with genital infections and increased HIV acquisition. *AIDS* 23 309–317. 10.1097/QAD.0b013e328321809c 19114868

[B64] Liu Y. Islam E. A. Jarvis G. A. Gray-Owen S. D. Russell M. W. (2012). *Neisseria gonorrhoeae* selectively suppresses the development of Th1 and Th2 cells, and enhances Th17 cell responses, through TGF-beta-dependent mechanisms. *Mucosal Immunol.* 5 320–331. 10.1038/mi.2012.12 22354319 PMC3328619

[B65] Liu Y. Liu W. Russell M. W. (2014). Suppression of host adaptive immune responses by *Neisseria gonorrhoeae*: role of interleukin 10 and type 1 regulatory T cells. *Mucosal Immunol.* 7 165–176. 10.1038/mi.2013.36 23757303 PMC3812424

[B66] Liu Y. Perez J. Hammer L. A. Gallagher H. C. De Jesus M. Egilmez N. K. (2018). Intravaginal Administration of Interleukin 12 during genital gonococcal infection in mice induces immunity to Heterologous Strains of *Neisseria gonorrhoeae*. *mSphere* 3:e00421-17. 10.1128/mSphere.00421-17 29404418 PMC5793040

[B67] Malott R. J. Keller B. O. Gaudet R. G. Hobbs J. L. McCaw S. E. Moraes T. F. (2013). *Neisseria gonorrhoeae*-derived heptose elicits an innate immune response and drives HIV-1 expression. *Proc. Natl. Acad. Sci.* 110 10234–10239. 10.1073/pnas.1303738110 23733950 PMC3690901

[B68] Martin-Moreno A. Munoz-Fernandez M. A. (2019). Dendritic cells, the double agent in the war against HIV-1. *Front. Immunol.* 10:2485. 10.3389/fimmu.2019.02485 31708924 PMC6820366

[B69] Mbonye U. Karn J. (2017). The Molecular Basis for Human Immunodeficiency Virus Latency. *Ann. Rev. Virol.* 4 261–285. 10.1146/annurev-virology-101416-041646 28715973

[B70] McGee Z. A. Gregg C. R. Johnson A. P. Kalter S. S. Taylor-Robinson D. (1990). The evolutionary watershed of susceptibility to gonococcal infection. *Microb. Pathog.* 9 131–139. 10.1016/0882-4010(90)90087-7 2126057

[B71] McGee Z. A. Stephens D. S. Hoffman L. H. Schlech W. F. III Horn R. G. (1983). Mechanisms of mucosal invasion by pathogenic Neisseria. *Rev. Infect. Dis.* 5(Suppl. 4), S708–S714. 6415784 10.1093/clinids/5.supplement_4.s708

[B72] McKinnon L. R. Nyanga B. Kim C. J. Izulla P. Kwatampora J. Kimani M. (2015). Early HIV-1 infection is associated with reduced frequencies of cervical Th17 cells. *J. Acquir. Immune Defic. Syndr.* 68 6–12. 10.1097/QAI.0000000000000389 25296095

[B73] McSheffrey G. G. Gray-Owen S. D. (2015). “Neisseria gonorrhoeae,” in *Molecular Medical Microbiology*, 2nd Edn, eds Tang Y.-W. Sussman M. Liu D. Poxton I. Schwartzman J. (Waltham, MA: Academic Press).

[B74] Melly M. A. McGee Z. A. Rosenthal R. S. (1984). Ability of monomeric peptidoglycan fragments from *Neisseria gonorrhoeae* to damage human fallopian-tube mucosa. *J. Infect. Dis.* 149 378–386. 10.1093/infdis/149.3.378 6425421

[B75] Meltzer M. S. Nakamura M. Hansen B. D. Turpin J. A. Kalter D. C. Gendelman H. E. (1990). Macrophages as susceptible targets for HIV infection, persistent viral reservoirs in tissue, and key immunoregulatory cells that control levels of virus replication and extent of disease. *AIDS Res. Hum. Retrovir.* 6 967–971. 10.1089/aid.1990.6.967 2223243

[B76] Milivojevic M. Dangeard A. S. Kasper C. A. Tschon T. Emmenlauer M. Pique C. (2017). ALPK1 controls TIFA/TRAF6-dependent innate immunity against heptose-1,7-bisphosphate of gram-negative bacteria. *PLoS Pathog.* 13:e1006224. 10.1371/journal.ppat.1006224 28222186 PMC5336308

[B77] Mirmonsef P. Krass L. Landay A. Spear G. T. (2012). The role of bacterial vaginosis and trichomonas in HIV transmission across the female genital tract. *Curr. HIV Res.* 10 202–210. 10.2174/157016212800618165 22384839 PMC3788616

[B78] Mlisana K. Naicker N. Werner L. Roberts L. van Loggerenberg F. Baxter C. (2012). Symptomatic vaginal discharge is a poor predictor of sexually transmitted infections and genital tract inflammation in high-risk women in South Africa. *J. Infect. Dis.* 206 6–14. 10.1093/infdis/jis298 22517910 PMC3490689

[B79] Morales P. Reyes P. Vargas M. Rios M. Imarai M. Cardenas H. (2006). Infection of human fallopian tube epithelial cells with *Neisseria gonorrhoeae* protects cells from tumor necrosis factor alpha-induced apoptosis. *Infect. Immun.* 74 3643–3650. 10.1128/IAI.00012-06 16714596 PMC1479248

[B80] Mosleh I. M. Boxberger H. J. Sessler M. J. Meyer T. F. (1997). Experimental infection of native human ureteral tissue with *Neisseria gonorrhoeae*: adhesion, invasion, intracellular fate, exocytosis, and passage through a stratified epithelium. *Infect. Immun.* 65 3391–3398. 10.1128/iai.65.8.3391-3398.1997 9234803 PMC175480

[B81] Muenzner P. Bachmann V. Zimmermann W. Hentschel J. Hauck C. R. (2010). Human-restricted bacterial pathogens block shedding of epithelial cells by stimulating integrin activation. *Science* 329 1197–1201. 10.1126/science.1190892 20813953

[B82] Nazli A. Chan O. Dobson-Belaire W. N. Ouellet M. Tremblay M. J. Gray-Owen S. D. (2010). Exposure to HIV-1 directly impairs mucosal epithelial barrier integrity allowing microbial translocation. *PLoS Pathog.* 6:e1000852. 10.1371/journal.ppat.1000852 20386714 PMC2851733

[B83] Oeckinghaus A. Hayden M. S. Ghosh S. (2011). Crosstalk in NF-kappaB signaling pathways. *Nat. Immunol.* 12 695–708. 10.1038/ni.2065 21772278

[B84] Patel P. Borkowf C. B. Brooks J. T. Lasry A. Lansky A. Mermin J. (2014). Estimating per-act HIV transmission risk: a systematic review. *AIDS* 28 1509–1519. 10.1097/QAD.0000000000000298 24809629 PMC6195215

[B85] Porter E. Yang H. Yavagal S. Preza G. C. Murillo O. Lima H. (2005). Distinct defensin profiles in *Neisseria gonorrhoeae* and *Chlamydia trachomatis* urethritis reveal novel epithelial cell-neutrophil interactions. *Infect. Immun.* 73 4823–4833. 10.1128/iai.73.8.4823-4833.2005 16040996 PMC1201278

[B86] Prendergast A. Prado J. G. Kang Y. H. Chen F. Riddell L. A. Luzzi G. (2010). HIV-1 infection is characterized by profound depletion of CD161+ Th17 cells and gradual decline in regulatory T cells. *AIDS* 24 491–502. 10.1097/QAD.0b013e3283344895 20071976

[B87] Pridmore A. C. Jarvis G. A. John C. M. Jack D. L. Dower S. K. Read R. C. (2003). Activation of toll-like receptor 2 (TLR2) and TLR4/MD2 by *Neisseria* is independent of capsule and lipooligosaccharide (LOS) sialylation but varies widely among LOS from different strains. *Infect. Immun.* 71 3901–3908. 10.1128/iai.71.7.3901-3908.2003 12819075 PMC161978

[B88] Quinn T. C. Wawer M. J. Sewankambo N. Serwadda D. Li C. Wabwire-Mangen F. (2000). Viral Load and Heterosexual Transmission of Human Immunodeficiency Virus Type 1. *N. Engl. J. Med.* 342 921–929. 10.1056/NEJM200003303421303 10738050

[B89] Ramsey M. E. Woodhams K. L. Dillard J. P. (2011). The gonococcal genetic island and type IV secretion in the pathogenic Neisseria. *Front. Microbiol.* 2:61. 10.3389/fmicb.2011.00061 21833316 PMC3153036

[B90] Rapista A. Ding J. Benito B. Lo Y.-T. Neiditch M. B. Lu W. (2011). Human defensins 5 and 6 enhance HIV-1 infectivity through promoting HIV attachment. *Retrovirology* 8:45. 10.1186/1742-4690-8-45 21672195 PMC3146398

[B91] Rawle D. J. Harrich D. (2018). Toward the “unravelling” of HIV: host cell factors involved in HIV-1 core uncoating. *PLoS Pathog.* 14:e1007270. 10.1371/journal.ppat.1007270 30286189 PMC6171947

[B92] Rodger A. J. Cambiano V. Bruun T. Vernazza P. Collins S. Degen O. (2019). Risk of HIV transmission through condomless sex in serodifferent gay couples with the HIV-positive partner taking suppressive antiretroviral therapy (PARTNER): final results of a multicentre, prospective, observational study. *Lancet* 393 2428–2438. 10.1016/S0140-6736(19)30418-0 31056293 PMC6584382

[B93] Rowley J. Vander Hoorn S. Korenromp E. Low N. Unemo M. Abu-Raddad L. J. (2019). Chlamydia, gonorrhoea, trichomoniasis and syphilis: global prevalence and incidence estimates, 2016. *Bull. World Health Organ.* 97 548–562. 10.2471/BLT.18.228486 31384073 PMC6653813

[B94] Royce R. A. Sena A. Cates W. Jr. Cohen M. S. (1997). Sexual transmission of HIV. *N. Engl. J. Med.* 336 1072–1078. 10.1056/NEJM199704103361507 9091805

[B95] Sadarangani M. Pollard A. J. Gray-Owen S. D. (2011). Opa proteins and CEACAMs: pathways of immune engagement for pathogenic *Neisseria*. *FEMS Microbiol. Rev.* 35 498–514. 10.1111/j.1574-6976.2010.00260.x 21204865

[B96] Salit I. E. (1982). The differential susceptibility of gonococcal opacity variants to sex hormones. *Can. J. Microbiol.* 28 301–306. 10.1139/m82-044 6805930

[B97] Schellenberg J. J. Card C. M. Ball T. B. Mungai J. N. Irungu E. Kimani J. (2012). Bacterial vaginosis, HIV serostatus and T-cell subset distribution in a cohort of East African commercial sex workers: retrospective analysis. *AIDS* 26 387–393. 10.1097/QAD.0b013e32834ed7f0 22095193

[B98] Shen R. Richter H. E. Clements R. H. Novak L. Huff K. Bimczok D. (2009). Macrophages in vaginal but not intestinal mucosa are monocyte-like and permissive to human immunodeficiency virus type 1 Infection. *J. Virol.* 83 3258–3267. 10.1128/JVI.01796-08 19153236 PMC2655566

[B99] Sheung A. Rebbapragada A. Shin L. Y. Dobson-Belaire W. Kimani J. Ngugi E. (2008). Mucosal *Neisseria gonorrhoeae* coinfection during HIV acquisition is associated with enhanced systemic HIV-specific CD8 T-cell responses. *AIDS* 22 1729–1737. 10.1097/QAD.0b013e32830baf5e 18753933

[B100] Sintsova A. Sarantis H. Islam E. A. Sun C. X. Amin M. Chan C. H. (2014). Global analysis of neutrophil responses to *Neisseria gonorrhoeae* reveals a self-propagating inflammatory program. *PLoS Pathog.* 10:e1004341. 10.1371/journal.ppat.1004341 25188454 PMC4154863

[B101] Smed-Sorensen A. Lore K. Vasudevan J. Louder M. K. Andersson J. Mascola J. R. (2005). Differential susceptibility to human immunodeficiency virus type 1 infection of myeloid and plasmacytoid dendritic cells. *J. Virol.* 79 8861–8869. 10.1128/JVI.79.14.8861-8869.2005 15994779 PMC1168781

[B102] Swanson J. Barrera O. Sola J. Boslego J. (1988). Expression of outer membrane protein II by gonococci in experimental gonorrhea. *J. Exp. Med.* 168 2121–2130. 3143800 10.1084/jem.168.6.2121 PMC2189168

[B103] Taylor B. D. Ness R. B. Darville T. Haggerty C. L. (2011). Microbial correlates of delayed care for pelvic inflammatory disease. *Sex Transm. Dis.* 38 434–438. 10.1097/OLQ.0b013e3181ffa7c7 21124257 PMC3657731

[B104] Thibault S. Fromentin R. Tardif M. R. Tremblay M. J. (2009). TLR2 and TLR4 triggering exerts contrasting effects with regard to HIV-1 infection of human dendritic cells and subsequent virus transfer to CD4+ T cells. *Retrovirology* 6:42. 10.1186/1742-4690-6-42 19419540 PMC2691729

[B105] Tugizov S. (2016). Human immunodeficiency virus-associated disruption of mucosal barriers and its role in HIV transmission and pathogenesis of HIV/AIDS disease. *Tissue Barriers* 4:e1159276. 10.1080/21688370.2016.1159276 27583187 PMC4993574

[B106] UNAIDS (2019). *FACT SHEET – Global AIDS Update 2019.* Geneva: UNAIDS.

[B107] Unemo M. Seifert H. S. Hook E. W. III Hawkes S. Ndowa F. Dillon J. R. (2019). Gonorrhoea. Nature Reviews. *Dis. Prim.* 5:79. 10.1038/s41572-019-0128-6 31754194

[B108] van Vliet S. J. Steeghs L. Bruijns S. C. M. Vaezirad M. M. Snijders Blok C. Arenas Busto J. A. (2009). Variation of *Neisseria gonorrhoeae* lipooligosaccharide directs dendritic cell–induced T Helper Responses. *PLoS Pathog.* 5:e1000625. 10.1371/journal.ppat.1000625 19834553 PMC2757725

[B109] Virji M. (2009). Pathogenic neisseriae: surface modulation, pathogenesis and infection control. *Nat. Rev. Microbiol.* 7 274–286. 10.1038/nrmicro2097 19287450

[B110] Wang L. C. Yu Q. Edwards V. Lin B. Qiu J. Turner J. R. (2017). *Neisseria gonorrhoeae* infects the human endocervix by activating non-muscle myosin II-mediated epithelial exfoliation. *PLoS Pathog.* 13:e1006269. 10.1371/journal.ppat.1006269 28406994 PMC5391109

[B111] Wi T. Lahra M. M. Ndowa F. Bala M. Dillon J. R. Ramon-Pardo P. (2017). Antimicrobial resistance in *Neisseria gonorrhoeae*: global surveillance and a call for international collaborative action. *PLoS Med.* 14:e1002344. 10.1371/journal.pmed.1002344 28686231 PMC5501266

[B112] Wiesenfeld H. C. Heine R. P. Krohn M. A. Hillier S. L. Amortegui A. A. Nicolazzo M. (2002). Association between elevated neutrophil defensin levels and endometritis. *J. Infect. Dis.* 186 792–797. 10.1086/342417 12198613

[B113] Xu S. X. Leontyev D. Kaul R. Gray-Owen S. D. (2018). *Neisseria gonorrhoeae* co-infection exacerbates vaginal HIV shedding without affecting systemic viral loads in human CD34+engrafted mice. *PLoS One* 13:e191672. 10.1371/journal.pone.0191672 29360873 PMC5779692

[B114] Yu Q. Chow E. M. McCaw S. E. Hu N. Byrd D. Amet T. (2013). Association of *Neisseria gonorrhoeae* Opa(CEA) with dendritic cells suppresses their ability to elicit an HIV-1-specific T cell memory response. *PLoS One* 8:e56705. 10.1371/journal.pone.0056705 23424672 PMC3570455

[B115] Yu Q. Wang L. C. Di Benigno S. Gray-Owen S. D. Stein D. C. Song W. (2019). *Neisseria gonorrhoeae* infects the heterogeneous epithelia of the human cervix using distinct mechanisms. *PLoS Pathog.* 15:e1008136. 10.1371/journal.ppat.1008136 31790511 PMC6907876

[B116] Zhang J. Li G. Bafica A. Pantelic M. Zhang P. Broxmeyer H. (2005). *Neisseria gonorrhoeae* Enhances Infection of Dendritic Cells by HIV Type 1. *J. Immunol.* 174 7995–8002. 10.4049/JIMMUNOL.174.12.7995 15944306

[B117] Zhou P. She Y. Dong N. Li P. He H. Borio A. (2018). Alpha-kinase 1 is a cytosolic innate immune receptor for bacterial ADP-heptose. *Nature* 561 122–126. 10.1038/s41586-018-0433-3 30111836

[B118] Zhu W. Tomberg J. Knilans K. J. Anderson J. E. McKinnon K. P. Sempowski G. D. (2018). Properly folded and functional PorB from *Neisseria gonorrhoeae* inhibits dendritic cell stimulation of CD4(+) T cell proliferation. *J. Biol. Chem.* 293 11218–11229. 10.1074/jbc.RA117.001209 29752412 PMC6052219

